# An interview with Nicola Maffulli, section editor for the musculoskeletal regenerative medicine section of *BMC Sports Science*, *Medicine and Rehabilitation*

**DOI:** 10.1186/2052-1847-5-14

**Published:** 2013-06-25

**Authors:** Nicola Maffulli

**Affiliations:** 1 Department of Musculoskeletal Surgery, University of Salerno School of Medicine and Surgery, Salerno, Italy; 2 Centre for Sports and Exercise Medicine, Queen Mary University of London, London, England

## 

Professor Nicola Maffulli is a Consultant Orthopaedic and Sports Injury Surgeon and has published over 800 peer reviewed articles in scientific journals and 12 books on Orthopaedic Surgery and in Sports Medicine. Professor Maffulli has a particular scientific interest in the physiopathology of sports injuries, including anterior cruciate ligament and tendon injuries, and great expertise in arthroscopic techniques of the knee and foot and ankle. He was Professor of Trauma and Orthopaedic Surgery at Keele University School of Medicine (2001–2008), Centre Lead and Professor of Sports and Exercise Medicine at Queen Mary University of London, Barts, and The London School of Medicine and Dentistry, London (2008–2013), and has now taken up the Chair in Musculoskeletal Disorders in Salerno University School of Medicine, Italy, while maintaining an Honorary Chair at Queen Mary University of London.

A keen athlete in his younger days, his boyhood dream of going to the Olympic Games eventually came true as in London 2013 he was the Field of Play doctor for the Olympic Wrestling Tournament, and led a group of seven orthopaedic surgeons to administer the Orthopaedic Foot and Ankle care for both the Olympics and the Paralympics. He is one of small handful of surgeons to have been awarded the FRCP (Fellowship of the Royal College of Physicians).

He is the Immediate Past President of the Sport and Exercise Section of the Royal Society of Medicine, and the President of the European Federation of the National Societies of Orthopaedic Sports Trauma (EFOST) for the period 2012–2014. Professor Maffulli has described more than 40 new surgical techniques in the field of knee, foot and ankle and sports surgery, and many have now become standard practice in these fields.

In this interview, we explore the field of musculoskeletal regenerative medicine and find out what Professor Maffulli would like to see submitted to his section.

## What does the musculoskeletal regenerative medicine field involve?

Regenerative medicine has been defined as a new field of applied medicine aimed at replacing or regenerating human cells, tissues, or organs to restore or establish normal function. In the musculoskeletal system, regenerative medicine involves muscles, ligaments, tendons, articular cartilage, menisci and bone. There is an additional complication with regenerative medicine in that all these new tissues have to be innervated and appropriately vascularised. This is a real challenge for the scientists in the field, as the procedures can often be quite complex in connecting up these new tissues properly.

## What is the most common mechanism of injury in sport?

No one single mechanism is responsible. Broadly speaking, we see acute injuries due to trauma, overuse injuries from excessive prolonged training, and chronic injuries when an injury has been underestimated and the athlete tries to return to sport too early. Often, these mechanisms of injury are avoidable and link back to athletes being aware of their body’s limitations and what they can and should be achieving with training.

## What have been the main advances in musculoskeletal regenerative medicine over the last few years and where are we today?

The field is still in its infancy and while there have been some promising reports, these haven’t yet translated into clinical trials and treatments. The future is bright, though, and inevitably things are happening. At present, there are some preliminary studies which show that for example, stem cells can be successfully added to the standard management of osteo-articular injuries, but routine use is still far away. Researchers are becoming more imaginative, and marrying several fields: tissue engineering, molecular biology, and genetics. What appears to be a necessity is to have greater inter-disciplinary communication, and to be able to span medicine, biology and engineering.

## Which tissues have been shown to particularly benefit from stem cell therapy?

Bone and cartilage have attracted the most interest. Some clinical studies are available, and await to be repeated by independent centres. Muscles, ligaments and tendons are probably the ultimate frontier in this field, especially given how common injuries to these structures are, and how troublesome and unpredictable their management is.

## Are there any particular injuries that are especially difficult to manage?

Yes. Chronic overuse injuries of the soft tissues are the most challenging. This is probably because we have not yet learnt how best to cope with them appropriately and this is something which requires ongoing work and research. In this respect, these modern techniques of stem cell research and tissue engineering may constitute a real revolution.

## What are the main problems or challenges with stem cell therapy and how can research contribute to meeting these challenges?

Stem cell therapy has had a lot of hype recently, but little research output and medical applications have emerged as of yet. While some studies have been conducted, those that have been especially well-performed have unfortunately been singularly negative. We are still struggling, but many groups are undertaking groundbreaking work and so the future looks positive. Eventually, we shall find the right cell and the right stimuli, and then stem cell therapy will be able to be widely used in sports medicine for the treatment of serious injuries and for the improvement of athlete strength and performance. Most of this work is expensive, and therefore its application is still limited to laboratory studies. Nevertheless, the programme, for example, in Pittsburgh, USA led by Professor Fu and his group, is state of the art, with great opportunities for translation into everyday clinical practice.

## As a clinician in the field, what do you think is the most important aspect to consider when treating athletes, both now and in the future?

A major issue is to make athletes understand that healing after injury takes time. From my viewpoint, the most common cause of failure of a well-performed treatment is attempts at early return to sport. To tackle this, regular contact with a sports practitioner is essential alongside stressing the importance of appropriate rest and recovery, ensuring that the body is stronger for training and competing.

## Where are we with helping athletes to effectively regain their full potential after injury?

We are now able to perform many surgical procedures in a less invasive fashion, and we are perfecting our rehabilitation techniques, resulting in reduced injury time. If we were able to harvest the potential of regenerative medicine, we would be able to make a real breakthrough!

## What will the new *BMC Sports Science*, *Medicine and Rehabilitation* bring to the sports medicine community?

This is a new and exciting opportunity to spread the word. The open access publishing model will allow everybody to read original research in the field, and will allow researchers to publish under one single banner. The importance of the visibility that open access guarantees in this key area of research cannot be overstated, as any new discovery can be immediately disseminated throughout the sports medicine community and follow-up studies can be put together.

## What kind of manuscripts would you like to see submitted to your section?

I am particularly interested in manuscripts on the topic of tissue engineering, gene expression, and novel techniques to optimise musculoskeletal cell cultures. The field of molecular and cellular modelling is still in its infancy, but manuscripts in these areas would be equally welcome in the section as they are important and growing areas of research. We welcome original research, commentaries and reviews on all these topics for consideration for peer review.

## Pre-publication history

The pre-publication history for this paper can be accessed here:

http://www.biomedcentral.com/2052-1847/5/14/prepub

